# Initial experience of cardiac T_1_ρ mapping at 0.55 T: Continuous wave versus adiabatic spin‐lock preparation pulses

**DOI:** 10.1002/mrm.30582

**Published:** 2025-05-20

**Authors:** Dongyue Si, Michael G. Crabb, Simon J. Littlewood, Karl P. Kunze, Juliet Varghese, Katherine Binzel, Mahmood Khan, Orlando P. Simonetti, Claudia Prieto, René M. Botnar

**Affiliations:** ^1^ School of Biomedical Engineering and Imaging Sciences King's College London London UK; ^2^ MR Research Collaborations Siemens Healthcare Limited Camberley UK; ^3^ Department of Biomedical Engineering The Ohio State University Columbus Ohio USA; ^4^ Dorothy M. Davis Heart and Lung Research Institute The Ohio State University Columbus Ohio USA; ^5^ Department of Emergency Medicine The Ohio State University Columbus Ohio USA; ^6^ Division of Cardiovascular Medicine, Department of Internal Medicine The Ohio State University Columbus Ohio USA; ^7^ Department of Radiology The Ohio State University Columbus Ohio USA; ^8^ School of Engineering Pontificia Universidad Católica de Chile Santiago Chile; ^9^ Millennium Institute for Intelligent Healthcare Engineering Santiago Chile; ^10^ Institute for Biological and Medical Engineering Pontificia Universidad Católica de Chile Santiago Chile; ^11^ Institute for Advanced Study Technical University of Munich Garching Germany

**Keywords:** adiabatic pulses, low‐field, myocardial tissue characterization, spin‐lock, T_1_ρ mapping

## Abstract

**Purpose:**

To propose and validate a cardiac T_1_ρ mapping sequence at 0.55 T comparing continuous‐wave and adiabatic spin‐lock (SL) preparation pulses.

**Methods:**

The proposed 2D sequence acquires four single‐shot balanced SSFP readout images with differing contrasts in a single breath‐hold. The first three images are prepared with T_1_ρ preparation pulses with different durations, while the last image uses a saturation pulse immediately before data acquisition. The T_1_ρ map is calculated using a 3‐parameter fitting method. Bloch equation simulations were performed to optimize the parameters of the adiabatic‐SL pulses. Phantom studies and in vivo experiments in 10 healthy volunteers, a porcine myocardial infarction model, and a patient with suspected hypertrophic cardiomyopathy were performed to validate the performance of the proposed adiabatic T_1_ρ (T_1_ρ_Ad_) mapping in comparison with conventional continuous‐wave T_1_ρ (T_1_ρ_CW_) mapping.

**Results:**

The adiabatic‐SL pulse with simulation‐optimized parameters demonstrated robust performance despite B_0_ and B_1_ field inhomogeneities. Phantom T_1_ρ_CW_ and T_1_ρ_Ad_ mapping exhibited comparable precision. In vivo experiments on healthy volunteers showed that myocardial T_1_ρ_Ad_ is higher than T_1_ρ_CW_ (106.1 ± 7.1 vs. 47.0 ± 5.1 ms, *p* < 0.01) with better precision (11.4% ± 2.6% vs. 14.5% ± 2.1%, *p* < 0.01) and less spatial variation (10.9% ± 3.0% vs. 14.4% ± 3.4%, *p* < 0.01). Both T_1_ρ_CW_ and T_1_ρ_Ad_ mapping agreed with late gadolinium enhancement findings in the porcine model and the patient, and exhibited improved contrast compared to T_1_ and T_2_ mapping.

**Conclusion:**

Both T_1_ρ_CW_ and T_1_ρ_Ad_ are promising for non‐contrast detection of various cardiomyopathies at 0.55 T, but T_1_ρ_Ad_ demonstrates better spatial uniformity than T_1_ρ_CW_.

## 1 INTRODUCTION

Cardiovascular magnetic resonance (CMR) parametric mapping can provide quantitative myocardial tissue characterization for various cardiac diseases.^1^ In addition to commonly used T_1_ and T_2_ relaxation parameters, T_1_ρ relaxation produced by spin‐lock (SL) preparation is a promising alternative for non‐contrast myocardial scar assessment,^2^, ^3^, ^4^, ^5^ feasible for the detection of various cardiomyopathies including chronic myocardial infarction (MI) and hypertrophic cardiomyopathy (HCM).^6^, ^7^, ^8^, ^9^ Native T_1_ρ mapping can provide findings consistent with late gadolinium enhancement (LGE),^6^, ^7^ and demonstrates higher differences between infarcted and remote myocardium than T_1_ and T_2_ mapping.^8^, ^10^

At present, T_1_ρ mapping has mostly been performed and validated at routine field strengths of 1.5 T or 3 T.^2^ As T_1_ρ preparation is commonly implemented with continuous wave (CW) SL pulses, T_1_ρ measurements could be sensitive to B_0_ and B_1_ field inhomogeneities especially at higher field strength,^11^ although different optimization methods have been proposed.^4^, ^11^, ^12^, ^13^, ^14^ Low‐field is particularly appealing for T_1_ρ mapping given the advantages of reduced field inhomogeneities.^15^, ^16^ More recently, commercial low‐cost low‐field MRI scanners have become available for clinical use, which have the potential to improve the accessibility of MRI.^16^, ^17^ Therefore, there has been increased interest in developing cardiac T_1_ρ mapping sequences at low‐field.

However, the experience with T_1_ρ mapping is currently very limited at low‐field.^18^ Preliminary experiments of a 3D whole‐heart joint T_1_/T_1_ρ mapping sequence have been performed on a commercial 0.55 T scanner.^19^ The limited power of the RF amplifier of this system necessitated a relatively low SL amplitude of 175 Hz for T_1_ρ preparation using CW‐SL, which may have limited T_1_ρ dispersion.^19^ The hardware limitations, therefore, make it challenging to implement T_1_ρ mapping with CW‐SL pulses at commercially available low‐field scanners. As an alternative, adiabatic‐SL pulses using adiabatic full passage pulses have been proposed to generate T_1_ρ contrast, which have been demonstrated to be more robust to field inhomogeneities than CW‐SL at 3 T.^20^, ^21^, ^22^ Furthermore, adiabatic‐SL pulses have reduced peak RF power requirements, making it possible to achieve a higher maximum SL amplitude than CW‐SL with the same hardware limitations.^23^

In this study, we sought to develop and validate a cardiac T_1_ρ mapping sequence at 0.55 T and compare the performance of CW‐SL and adiabatic‐SL preparation pulses. Four 2D images with different contrasts are acquired in a single breath‐hold, and a T_1_ρ map is then calculated pixel‐by‐pixel from the four images using a 3‐parameter fitting method. Bloch equation simulations were performed to optimize the parameters of the adiabatic‐SL pulses. Phantom studies and in vivo experiments in healthy volunteers, a porcine model of myocardial infarction, and a patient with hypertrophic cardiomyopathy were performed to validate the performance of the proposed adiabatic T_1_ρ (T_1_ρ_Ad_) mapping in comparison with conventional CW T_1_ρ (T_1_ρ_CW_) mapping.

## 2 METHODS

### 2.1 Pulse sequence design

**FIGURE 1:**
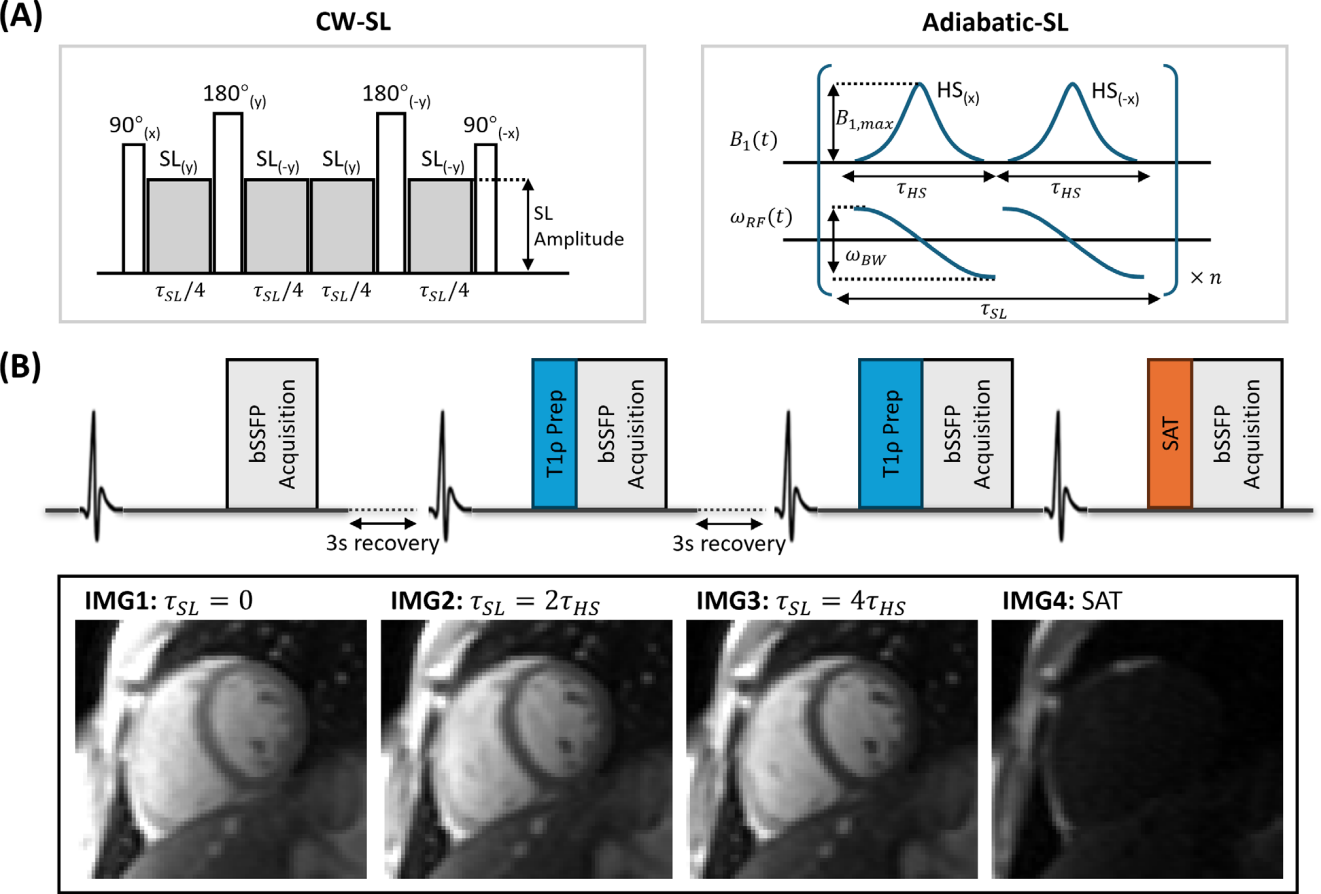
Pulse sequence framework. (A) Continuous wave (CW) and adiabatic spin‐lock (SL) T_1_ρ preparation pulses used in this study. Adiabatic‐SL pulse is comprised of an even number (*n*) of adiabatic full passage hyperbolic‐secant (HS) pulses with opposite phases, which has a duration of $\tau_{\mathrm{SL}}=n\times \tau_{\mathrm{HS}}$. (B) Diagram and representative images of the proposed adiabatic T_1_ρ mapping sequence. Four images with different contrasts are acquired with single‐shot balanced SSFP (bSSFP) in a single breath‐hold. The first three images are prepared with T_1_ρ preparation pulses with different durations ($\tau_{\mathrm{SL}}$ = 0, 2 $\tau_{\mathrm{HS}}$, 4 $\tau_{\mathrm{HS}}$), while the last image uses saturation (SAT) pulse immediately before data acquisition. Recovery periods of 3 s are used before the acquisition of the second and the third images, leading to a total scan time of approximately 10 s.

The SL pulses used for T_1_ρ preparation in this study are shown in Figure 1A. The reference CW‐SL is implemented with a totally balanced SL preparation module using two 180° refocusing pulses with opposite phases, and SL pulses with alternating phases, which has shown an improved performance regarding B_0_ and B_1_ field inhomogeneities.^5^, ^24^ Adiabatic‐SL consists of multiple pairs of hyperbolic‐secant (HS) 180° refocusing pulses with opposite phases, each having a duration of $\tau_{\mathrm{HS}}$, and is defined by the following amplitude ($B_{1}$) and frequency ($\omega_{\mathrm{RF}}$) modulation functions:

$$B_{1}\left(t\right)=B_{1,\max}\text{sech}\left(\beta t\right)$$

$$\omega_{\mathrm{RF}}\left(t\right)-\omega_{0}=-\mu \beta \tanh\left(\beta t\right),$$

where $t\in \left[-\frac{\tau_{\mathrm{HS}}}{2},\frac{\tau_{\mathrm{HS}}}{2}\right]$, $B_{1,\max}$ is the peak $B_{1}$ amplitude, β is a truncation factor characterizing the width of the pulse bell, $\omega_{0}$ is the Larmor frequency, and $\omega_{\mathrm{BW}}=2\mu \beta$ defines the bandwidth of $\omega_{\mathrm{RF}}\left(t\right)$.

The proposed T_1_ρ mapping research sequence is shown in Figure 1B. Four electrocardiogram‐triggered single‐shot balanced SSFP (bSSFP) readout images with different contrasts are acquired in a single breath‐hold. The first three images are prepared with T_1_ρ preparation pulses with different durations ($\tau_{\mathrm{SL}}$), while the last image uses a saturation (SAT) pulse immediately before data acquisition. A pulse train SAT was used to achieve a good SAT efficiency.^25^ Idle periods of 3 s are inserted before the acquisition of the second and the third images to allow sufficient magnetization recovery and minimize the influence of preceding acquisitions, resulting in a total scan time of approximately 10 s. The four images are co‐registered inline using an efficient symmetric and inverse‐consistent deformable registration algorithm,^26^ and then used to calculate a T_1_ρ map pixel‐by‐pixel offline with a 3‐parameter fitting model, which has an improved performance than 2‐parameter and 3‐parameter fitting without the SAT‐prepared image.^27^

For T_1_ρ_CW_ mapping, four images were acquired with $\tau_{\mathrm{SL}}$ = 2, 30, 60 ms and SAT, using a SL amplitude of 150 Hz. For T_1_ρ_Ad_ mapping, four images with $\tau_{\mathrm{SL}}$ = 0, 2 $\tau_{\mathrm{HS}}$, 4 $\tau_{\mathrm{HS}}$, and SAT were acquired. Adiabatic‐SL pulse had the following parameters: $\tau_{\mathrm{HS}}$ = 15 ms, $B_{1,\max}=11.7\;\mu T$ ($\omega_{1,\max}=\gamma B_{1,\max}=$ 500 Hz). To optimize the other two parameters: $\omega_{\mathrm{BW}}$ and μ, numerical simulation of the related preparation efficiency was performed with Bloch equation. Candidate adiabatic‐SL pulses consisted of two HS pulses with different $\omega_{\mathrm{BW}}$ and μ, and the average preparation efficiency was calculated over the expected range of in vivo B_0_ and B_1_ inhomogeneities.^21^ Pilot experiments on three healthy volunteers were performed to estimate the field inhomogeneities at 0.55 T. Based on the results (Figure S1), B_0_ off‐resonance ranging from −100 to 100 Hz and B_1_ factor ranging from 0.7 to 1 were used in the simulation. $\omega_{\mathrm{BW}}$ and μ that achieved the maximum average preparation efficiency were selected for the following experiments in this study. To evaluate the performance of the proposed sequence for T_1_ρ mapping, additional simulation experiments are performed as detailed in Supporting Information.

All MR experiments were performed on a clinical 0.55 T MR scanner (MAGNETOM Free.Max, Siemens Healthineers) with an external electrocardiography monitoring system (Expression MR400, Philips Healthcare) using a 6‐channel chest array and a 9‐channel embedded posterior receiver coil. The human experiments were approved by the local institutional review board (REMAS 8700). Written informed consent was obtained from all subjects before imaging. Animal studies were approved by the local Institutional Animal Care and Use Committee (2012A00000019). Numerical simulations, image processing, and statistical analysis were implemented in MATLAB R2023a (The MathWorks).

### 2.2 Phantom experiments

Phantom experiments were performed on a standardized 1.5 T T1MES phantom to validate the performance of the proposed 2D T_1_ρ_Ad_ mapping sequence.^28^ Both T_1_ρ_CW_ and T_1_ρ_Ad_ mapping were performed and 2D T_1_ and T_2_ mapping were also acquired as references using MOLLI and T_2_‐prepared bSSFP research sequences, modified for use at 0.55 T, respectively.^29^, ^30^ The imaging parameters for all four research sequences are shown in Table S1. Relatively larger voxel size and slice thickness, and higher readout flip angles were used for imaging at low‐field compared to higher field.

For phantom data analysis, circular regions of interest (ROI) covering the center of each phantom vial were manually defined on every parametric map. Mean value and coefficient of variation (CV), the percentage of the standard deviation divided by the mean, of T_1_, T_2_, T_1_ρ_CW_, and T_1_ρ_Ad_ were calculated for the pixels within the ROI for each vial. As these relaxation parameters have completely different absolute values, only CVs were compared to evaluate the precision of measurements.

### 2.3 In vivo experiments

Ten healthy volunteers (7 males, 30 ± 4 years) were recruited for CMR scanning to validate the performance of the proposed T_1_ρ_Ad_ mapping sequence in comparison to the T_1_ρ_CW_ mapping sequence. The imaging parameters were the same as those used in phantom experiments (Table S1). Three short‐axis slices were acquired at the apex, middle, and base of the left ventricle. Five of 10 volunteers (3 males, 29 ± 4 years) repeated the T_1_ρ_CW_ and T_1_ρ_Ad_ mapping at middle short‐axis to evaluate the repeatability.

A swine (2 months old and weighing 31.3 kg) was also included in this study. CMR imaging was performed at 1‐week post MI procedure that involved a 90‐min occlusion followed by reperfusion of the left circumflex artery. Two‐dimensional sequences including MOLLI, T_2_‐prepared bSSFP, T_1_ρ_CW_, and T_1_ρ_Ad_ mapping were acquired before contrast, while phase sensitive inversion recovery (PSIR) LGE were acquired post‐contrast following administration of 0.2 mmol/kg of gadobutrol (GADOVIST, Bayer Healthcare), with previously described imaging protocols.^16^ The imaging parameters of MOLLI and T_2_‐prepared bSSFP in swine experiments were slightly different from those used in vivo, and the resolution was 1.7 × 1.7 mm^2^. Pre‐contrast T_1_, T_2_, T_1_ρ_CW_, and T_1_ρ_Ad_ mapping and post‐contrast PSIR LGE images were also acquired for a preliminary clinical validation in a 60‐year‐old male patient with suspected HCM.

For in vivo maps, the epicardial and endocardial contours of the left ventricle were manually segmented on each map and divided into 16 segments for analysis according to the American Heart Association (AHA) 17‐segment model (without apical segment).^31^ T_1_ρ_CW_ and T_1_ρ_Ad_ mean value ($\mu_{s,v}$) and CV (${\mathrm{CV}}_{s,v}$) were calculated in each AHA segment (s) for all healthy volunteers (v). The results averaged across all healthy volunteers (${\bar{\mu }}_{s},{\bar{\mathrm{CV}}}_{s}$) were visualized using the bull's‐eye plot, and ${\bar{\mathrm{CV}}}_{s}$ was used to evaluate the precision. In addition, the spatial variability of the T_1_ρ_CW_ and T_1_ρ_Ad_ for each subject was evaluated by segment‐wise CV ($\mathrm{sC}V_{v}$), which is calculated as the standard deviation divided by the mean of the results in all 16 segments ($\mathrm{sC}V_{v}=\frac{\mathrm{std}\left[\mu_{1,v},\mu_{2,v},\ldots ,\mu_{16,v}\right]}{\text{mean}\left[\mu_{1,v},\mu_{2,v},\ldots ,\mu_{16,v}\right]}$). For the five volunteers with repeated scans, the repeatability is evaluated by relative bias (RBS) in each AHA segment, which is calculated as the absolute bias between the two measurements divided by the mean value of the two measurements ($\mathrm{RB}S_{s,v}=\frac{|\mu_{s,v,1}-\mu_{s,v,2}|}{\text{mean}\left[\mu_{s,v,1},\mu_{s,v,2}\right]}$), and the results averaged across the five volunteers (${\bar{\mathrm{RBS}}}_{s}$) were visualized. A paired 2‐tailed Student's *t* test (α = 0.05) was used for statistical analyses of the differences between T_1_ρ_CW_ and T_1_ρ_Ad_ mapping results.

## 3 RESULTS

Simulation results for the selection of adiabatic SL pulse parameters are shown in Figure S2. As $\omega_{\mathrm{BW}}$ = 800 Hz and μ = 4 achieved the maximum average preparation efficiency over the interest region of B_0_ and B_1_ inhomogeneities, these parameters were used in phantom and in vivo studies.

### 3.1 Phantom experiments

**FIGURE 2:**
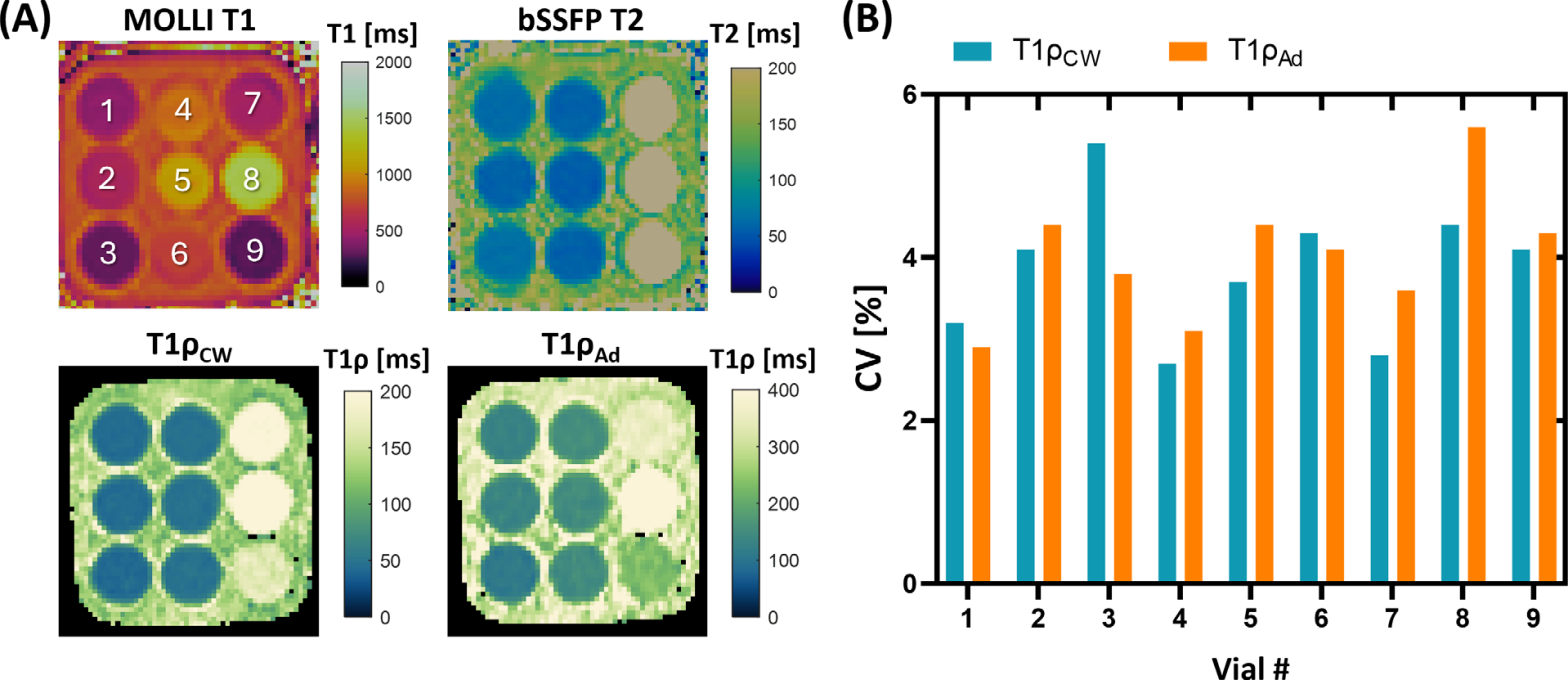
Phantom results. (A) T_1_, T_2_, T_1_ρ_CW_, and T_1_ρ_Ad_ maps acquired with 2D MOLLI, T_2_‐prepared balanced SSFP (bSSFP), and the proposed 2D T_1_ρ mapping sequences. (B) Coefficient of variation (CV) of the measured T_1_ρ_CW_ and T_1_ρ_Ad_ values for each phantom vial.

Phantom T_1_, T_2_, T_1_ρ_CW_, and T_1_ρ_Ad_ maps acquired with MOLLI, T_2_‐prepared bSSFP and the proposed sequence are shown in Figure 2A, with the related T_1_ρ_CW_ and T_1_ρ_Ad_ CV of each phantom vial shown in Figure 2B. The CV of T_1_ρ_Ad_ (4.0% ± 0.8%) are comparable with that of T_1_ρ_CW_ (3.9% ± 0.9%, *p* = 0.56). The detailed results of mean value and CV in each phantom vial are summarized in Table S2. T_1_ρ_Ad_ has higher CV than T_1_ρ_CW_ for phantom vials 7 to 9, which has longer T_1_ρ values mimicking blood. However, T_1_ρ_Ad_ is generally better than T_1_ρ_CW_ for phantom vials 1 to 6 with shorter T_1_ρ values mimicking myocardium.

### 3.2 Healthy volunteers

**FIGURE 3:**
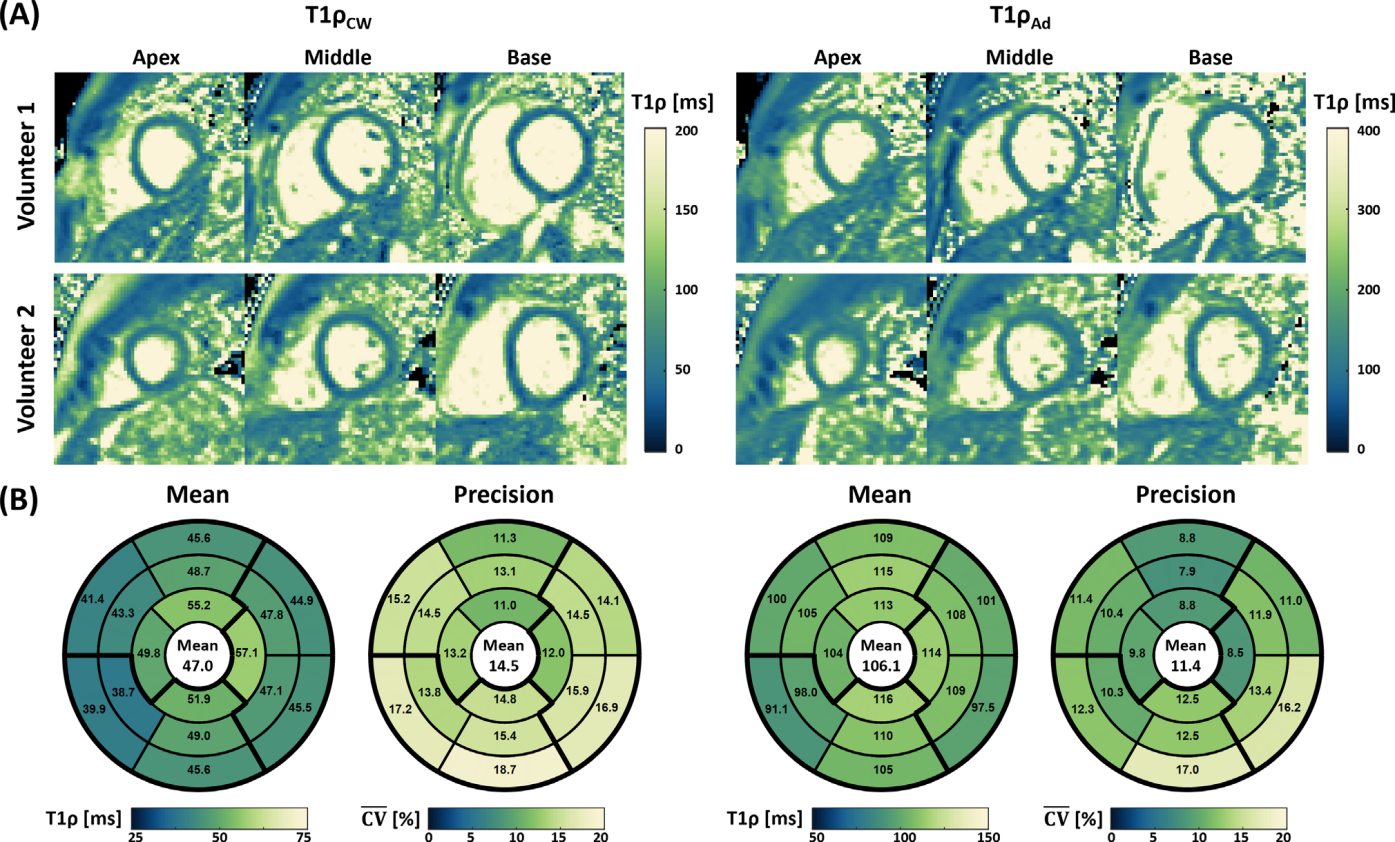
Healthy subject results. (A) Representative T_1_ρ_CW_ and T_1_ρ_Ad_ maps at apex, middle, and base short‐axis slices of two healthy volunteers acquired with the proposed sequence. (B) American Heart Association 16 segments bull's eye plots of myocardial T_1_ρ_CW_ and T_1_ρ_Ad_ mean values and coefficient of variations (CV) averaged across all 10 healthy volunteers. Myocardium T_1_ρ_Ad_ is significantly higher than myocardium T_1_ρ_CW_ (106.1 ± 7.1 vs. 47.0 ± 5.1 ms, *p* < 0.01), while CV of T_1_ρ_Ad_ is significantly lower than that of T_1_ρ_CW_ (11.4% ± 2.6% vs. 14.5% ± 2.1%, *p* < 0.01). The CVs in the inferior and base segments are slightly increased for both T_1_ρ_CW_ and T_1_ρ_Ad_ mapping, which could be because of the decreased SNR with the attenuation of coil sensitivity.

CMR imaging was successfully performed on all healthy volunteers. Figure S3 compares the T_2_ (T_1_ρ_CW_ at 0 Hz), T_1_ρ_CW_, and T_1_ρ_Ad_ maps of a healthy volunteer acquired with different sequences. Representative T_1_ρ_CW_ and T_1_ρ_Ad_ maps acquired from two healthy volunteers are shown in Figure 3A, demonstrating visually comparable image quality for left ventricle myocardium, although T_1_ρ_CW_ maps exhibit a slightly cleaner blood pool. Moreover, T_1_ρ_Ad_ demonstrates less spatial variation across the AHA segments than T_1_ρ_CW_ regardless of field inhomogeneities (Figure S4). Figure 3B shows the bull's‐eye plots of the myocardial T_1_ρ_CW_ and T_1_ρ_Ad_ mean values and CVs measured by the proposed sequence averaged across all healthy volunteers. Myocardial T_1_ρ_Ad_ values are significantly different from myocardial T_1_ρ_CW_ (106.1 ± 7.1 vs. 47.0 ± 5.1 ms, *p* < 0.01), whereas $\bar{\mathrm{CV}}$ of T_1_ρ_Ad_ is significantly lower than that of T_1_ρ_CW_ (11.4% ± 2.6% vs. 14.5% ± 2.1%, *p* < 0.01), indicating a better precision.^20^ In addition, sCV of T_1_ρ_Ad_ measurements are significantly lower than that of T_1_ρ_CW_ (10.9% ± 3.0% vs. 14.4% ± 3.4%, *p* < 0.01), showing less spatial variation over the segments across the heart. Furthermore, the repeatability of T_1_ρ_Ad_ mapping is slightly better than that of T_1_ρ_CW_ mapping with slightly lower $\bar{\mathrm{RBS}}$ (9.7% ± 3.5% vs. 10.5% ± 3.7%) as shown in Figure S5.

### 3.3 Porcine model and patient

**FIGURE 4:**
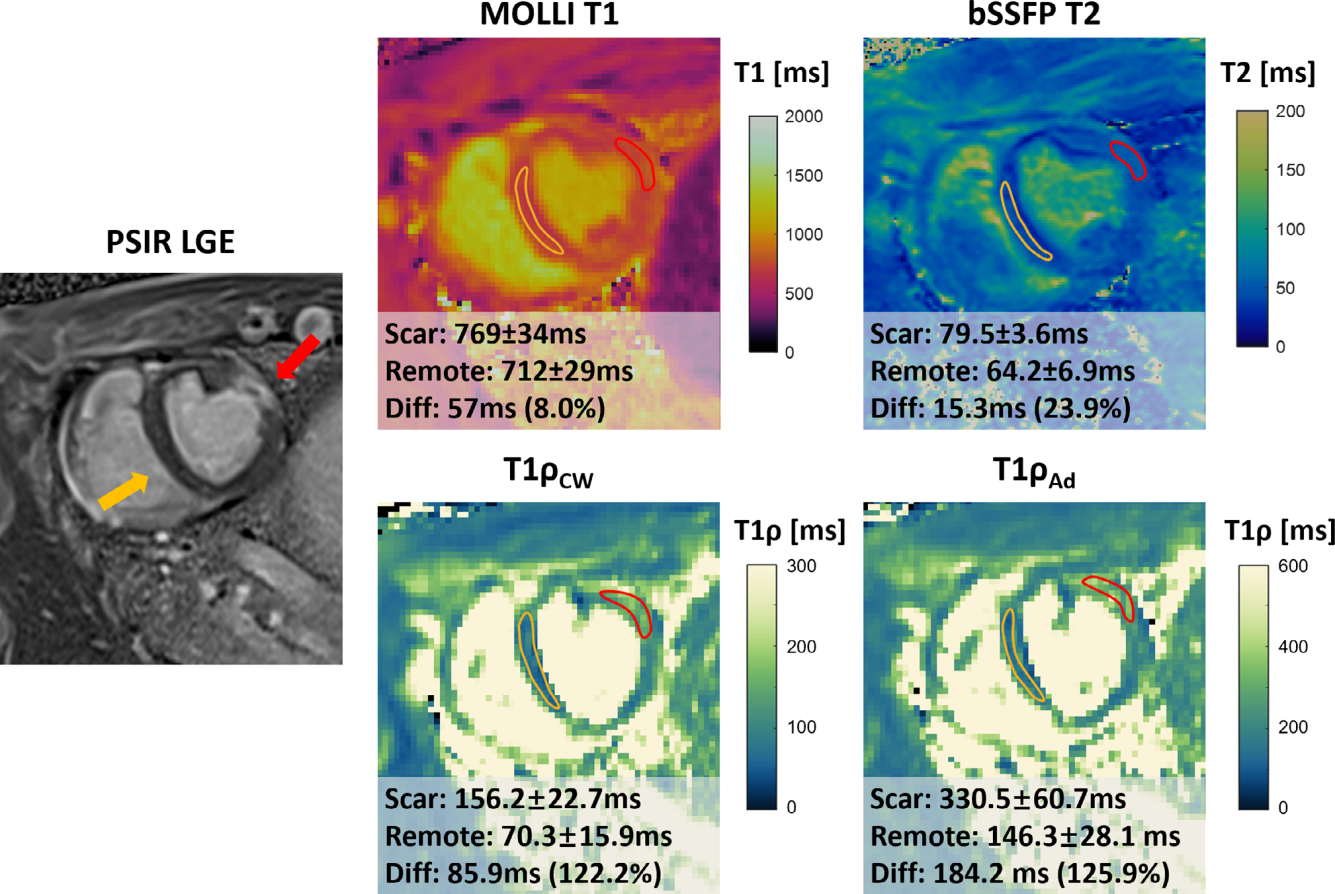
Representative 2D images acquired from the porcine model at 1‐week post myocardial infarction (MI) including late gadolinium enhancement (LGE) and T_1_, T_2_, T_1_ρ_CW_, and T_1_ρ_Ad_ maps with different sequences. LGE imaging presents enhancement in the infarcted area at the lateral segment. The tissue characterization results of MI scar (red arrow) and remote myocardium (yellow arrow) are shown in the corresponding parametric maps. T_1_ map shows slight increase in MI area compared with the remote myocardium, but T_2_, T_1_ρ_CW_, and T_1_ρ_Ad_ of MI area are all significantly increased compared to remote. bSSFP, balanced SSFP; PSIR, phase‐sensitive inversion recovery.

The pre‐contrast T_1_, T_2_, T_1_ρ_CW_, and T_1_ρ_Ad_ maps and LGE images acquired in the porcine model at 1‐week post MI are shown in Figure 4. LGE imaging demonstrates enhancement in the infarcted area in the lateral segment. The T_1_ map shows slightly alterations at the location of the MI area compared with the remote myocardium (769 ± 34 vs. 712 ± 29 ms, 8.0%), whereas T_2_ shows a distinct increase (79.5 ± 3.6 vs. 64.2 ± 6.9 ms, 23.9%) and T_1_ρ_CW_ and T_1_ρ_Ad_ are both significantly increased (156.2 ± 22.7 vs. 70.3 ± 15.9 ms, 122.2% and 330.5 ± 60.7 vs. 146.3 ± 28.1 ms, 125.9%). Both T_1_ρ_CW_ and T_1_ρ_Ad_ maps exhibit artifacts in the inferior segment, particularly where the myocardial wall is thin. These artifacts are primarily because of residual motion between images as this specific swine was in a relatively unstable status with heart rate around 120 bpm.

**FIGURE 5:**
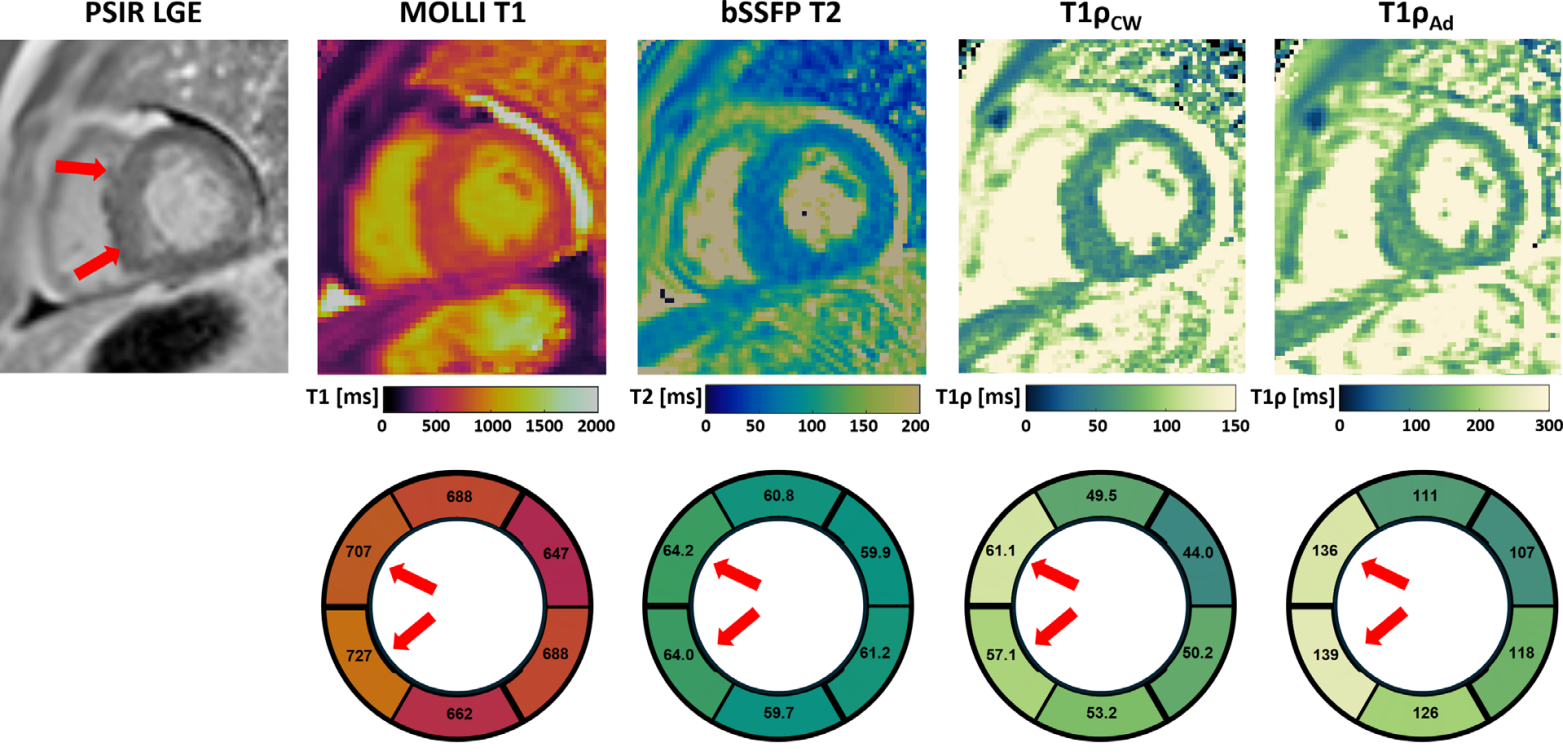
Representative 2D images of a patient with suspected hypertrophic cardiomyopathy including late gadolinium enhancement (LGE) and T_1_, T_2_, T_1_ρ_CW_, and T_1_ρ_Ad_ maps. LGE image shows patchy enhancement at the hypertrophied septum (red arrows). Corresponding American Heart Association analysis results of the maps are shown. T_1_, T_2_, T_1_ρ_CW_, and T_1_ρ_Ad_ are all elevated in the septal segments (red arrows) compared with the remote segment. bSSFP, balanced SSFP; PSIR, phase‐sensitive inversion recovery.

Images from the patient with suspected HCM are shown in Figure 5. Patchy enhancement can be seen in the hypertrophied septum in the LGE images. T_1_ and T_2_ are both elevated in the septal segments compared with the remote segments. T_1_ρ_CW_ and T_1_ρ_Ad_ maps also show consistent elevation of T_1_ρ_CW_ and T_1_ρ_Ad_ values in the septal segments.

## 4 DISCUSSION

In this study, we proposed a 2D cardiac T_1_ρ mapping sequence at the commercial 0.55 T system and compared the performance of CW‐SL and adiabatic‐SL pulses. The adiabatic‐SL pulse with parameters optimized in simulations demonstrates good performance despite field inhomogeneities. Phantom experiments showed that the T_1_ρ_CW_ and T_1_ρ_Ad_ mapping have comparable precision. In vivo experiments in healthy volunteers showed that myocardial T_1_ρ_Ad_ values were more spatially uniform and had greater precision than T_1_ρ_CW_. Preliminary validations in a porcine model of MI and one patient with HCM indicated that low‐field T_1_ρ_CW_ and T_1_ρ_Ad_ mapping are promising for the detection of both focal and patchy fibrosis.

To the best of our knowledge, this is the first study demonstrating the feasibility of T_1_ρ mapping using adiabatic‐SL pulses at 0.55 T. In comparison with conventional T_1_ρ_CW_ mapping, T_1_ρ_Ad_ mapping had two major advantages. First, adiabatic‐SL achieved an improved T_1_ρ dispersion than CW‐SL with a higher maximum SL amplitude (500 Hz vs. 150 Hz) on a commercial 0.55 T scanner. The mechanism of T_1_ρ relaxation with adiabatic‐SL is intrinsically different from conventional T_1_ρ relaxation with CW‐SL because of the time dependent amplitude and frequency modulation functions. As a result, myocardial T_1_ρ_Ad_ resulted in significantly higher T_1_ρ values compared with T_1_ρ_CW_ (106.1 ± 7.1 vs. 47.0 ± 5.1 ms, *p* < 0.01). These findings are consistent with previous studies of T_1_ρ_Ad_ mapping at 3 T and 1.5 T, showing a markedly higher T_1_ρ_Ad_ compared to T_1_ρ_CW._ ^21^, ^22^ The improved T_1_ρ dispersion can enhance the contrast between diseased and normal tissues as the range of T_1_ρ values across different tissues are increased.^8^ Our preliminary results in a porcine model of MI and a patient with HCM show slight improvements of contrast with T_1_ρ_Ad_ compared to T_1_ρ_CW_. This may be because the tissue T_1_ρ is closely related to histological changes, and increasing the SL amplitude to the kHz range may be beneficial for better probing molecular motional processes.^2^, ^3^ Further study with histological validation will be required to validate T_1_ρ dispersion. In conclusion, both T_1_ρ_CW_ and T_1_ρ_Ad_ mapping show improved contrast for diseased myocardium compared to T_1_ and T_2_ mapping, therefore, both demonstrate potential for disease detection at 0.55 T.

Second, as adiabatic‐SL is less sensitive to field inhomogeneities than CW‐SL, T_1_ρ_Ad_ mapping had better precision than T_1_ρ_CW_ mapping. Measurement precision is important for a robust and reproducible assessment in clinical cardiac applications.^32^ Here, the CV of myocardium T_1_ρ_Ad_ mapping (11.4% ± 2.6%) was significantly lower than that of T_1_ρ_CW_ (14.5% ± 2.1%), which is also lower than previously reported CV of T_1_ρ_Ad_ mapping at 1.5 T (13.62% ± 1.33%) and at 3 T (14.51% ± 3.71%). Although these studies were performed with different parameters and sequences and on different subject cohorts, the results indicate that despite the lower SNR at 0.55 T, the reduced field inhomogeneities resulted in fewer T_1_ρ signal variations and, therefore, better precision, making T_1_ρ mapping with adiabatic‐SL pulses a promising alternative for non‐contrast tissue characterization at 0.55 T.

It is worth noting that the T_1_ρ_Ad_ values can be related to the parameters ($B_{1,\max}$, $\tau_{\mathrm{HS}}$, $\omega_{\mathrm{BW}}$, and μ) of the adiabatic‐SL preparation and that the nominal value is an average result of the relaxation that occurs during the temporal evolution of the RF fields of the HS pulse.^20^ $\omega_{\mathrm{BW}}$ and μ were optimized with fixed $B_{1,\max}$ and $\tau_{\mathrm{HS}}$ in this study. $B_{1,\max}$ is limited by the peak B_1_ of the RF system, which was set to achieve a $\omega_{1,\max}$ of 500 Hz. The pulse duration $\tau_{\mathrm{HS}}$ is restricted by specific absorption rate (SAR) limits and the RF amplifier chain. Previous experiments at 3 T showed that longer $\tau_{\mathrm{HS}}$ improved T_1_ρ preparation performance.^21^ In theory, low‐field MRI with lower SAR levels allows for a longer SL pulse duration compared to high‐field MRI. Our preliminary experience at 0.55 T did not encounter SAR limitations. However, RF power constraints of the scanner caused sequence failure for $\tau_{\mathrm{HS}}$ durations longer than 20 ms. Therefore, $\tau_{\mathrm{HS}}$ of 15 ms was used. Future studies are warranted to determine if using a different combination of parameters may lead to a better performance for T_1_ρ_Ad_ mapping results.

This study has some limitations. First, the proposed sequence scheme may be improved in the future. The maximum $\tau_{\mathrm{SL}}$ is limited to 4 $\tau_{\mathrm{HS}}$ (60 ms) because of the RF power limitation, which may introduce bias in the measurement of longer T_1_ρ values. However, because of 3‐parameter fitting with a SAT image, it performs well for normal myocardium, as demonstrated by simulations (Figure S6) and in vivo results. Increasing the SL preparation time would require an upgrade of the RF hardware. A 2D breath‐hold acquisition was used, which may cause artifacts because of the residual motion between images. Free‐breathing non‐rigid motion‐corrected 3D whole heart imaging sequences may have the potentially to provide high‐resolution tissue characterization.^33^ Second, the performance of T_1_ρ_Ad_ mapping at low‐field for the detection of different cardiomyopathies requires additional validation. Our preliminary results showed that both T_1_ρ_CW_ and T_1_ρ_Ad_ mapping resulted in good agreement with LGE and demonstrated better contrast than conventional T_1_ and T_2_ mapping techniques especially for the porcine model at 1‐week after MI. Nevertheless, a larger cohort of patients and histological validation on porcine models would be needed for further comparisons between different parametric mapping techniques.

## 5 CONCLUSION

A cardiac T_1_ρ mapping sequence was proposed at 0.55 T, and the performance of CW‐SL and adiabatic‐SL pulses were compared. Preliminary validations in a porcine model and a patient indicated that both T_1_ρ_CW_ and T_1_ρ_Ad_ mapping are promising for non‐contrast detection of focal and diffuse tissue alterations such as observed in myocardial infarction and cardiomyopathies. However, the proposed T_1_ρ_Ad_ mapping technique had better precision and resulted in a more uniform appearance across the left ventricle myocardium than conventional T_1_ρ_CW_ mapping.

## CONFLICT OF INTEREST STATEMENT

Dr. Karl P. Kunze is an employee of Siemens Healthcare Limited.

## Supporting information


**Text S1.** Sequence simulation experiments.
**Table S1.** Imaging parameters of conventional 2D T1 and T2 mapping and the proposed T1ρ mapping sequences. bSSFP, balanced steady‐state free precession; MOLLI, modified Look‐Locker inversion recovery.
**Table S2.** Phantom T1, T2, T1ρ_CW_ and T1ρ_Ad_ in each vial. bSSFP, balanced steady‐state free precession; CV, coefficient of variation; MOLLI, modified Look‐Locker inversion recovery; SD, standard deviation.
**Figure S1.** B0 and B1 maps of three healthy volunteers at 0.55 T.
**Figure S2.** Simulation results for adiabatic spin‐lock (SL) pulse optimisation. (A) The average preparation efficiency of different candidate adiabatic‐SL pulses with different $\omega_{\mathrm{BW}}$ and μ. The optimal ($\omega_{\mathrm{BW}}$, μ) = (800 Hz, 4) is denoted with the dashed box. (B) Preparation efficiency profile of the optimized adiabatic‐SL pulse. The B0 and B1 design region (B0 off‐resonance range = [−100 100] Hz and B1 factor range = [0.7,1]) is illustrated with the dashed box.
**Figure S3.** bSSFP T2 mapping with 2‐parameter fitting in comparison with T1ρ_CW_ and T1ρ_Ad_ mapping with 3‐parameter fitting. Septal T2 and T1ρ values are shown below the images. T2 (T1ρ_cw_ at 0 Hz) map using the proposed sequence with 3‐parameter fitting (41.6 ± 6.8 ms) has lower myocardial T2 values than the bSSFP T2 map with 2‐parameter fitting (51.0 ± 4.8 ms). But T1ρ_cw_ at 150 Hz (47.3 ± 8.0 ms) is higher than T1ρ_cw_ at 0 Hz using the same 3‐parameter sequence scheme, showing the effect of T1ρ dispersion.
**Figure S4.** B0 and B1 field maps along with AHA plots of mean values and precision for T1ρ_CW_ and T1ρ_Ad_ for the same two volunteers in Figure 3A. Segment‐wise CV (sCV) was also calculated to demonstrate the spatial variability of the T1ρ_CW_ and T1ρ_Ad_ for each subject.
**Figure S5.** Repeatability of myocardial T1ρ_CW_ and T1ρ_Ad_ mapping at each AHA segment in the middle short‐axis slice averaged across all five healthy volunteers. RBS, relative bias.
**Figure S6.** Simulated T1ρ relative errors using different sampling schemes (A) and using the proposed sampling scheme for different T1 values (B).
