# Supplementary material for: Initial experience of cardiac T 1ρ mapping at 0.55 T: Continuous wave versus adiabatic spin‐lock preparation pulses
Source: Magn Reson Med. 2025 May 20;94(4):1644–53. doi: 10.1002/mrm.30582 (PMC12309885; doi:10.1002/mrm.30582)
Supplement: Supplementary file 1 — Text S1. Sequence simulation experiments. Table S1. Imaging parameters of conventional 2D T1 and T2 mapping and the proposed T1ρ mapping sequences. bSSFP, balanced steady‐state free precession; MOLLI, modified Look‐Locker inversion recovery. Table S2. Phantom T1, T2, T1ρCW and T1ρAd in each vial. bSSFP, balanced steady‐state free precession; CV, coefficient of variation; MOLLI, modified Look‐Locker inversion recovery; SD, standard deviation. Figure S1. B0 and B1 maps of three healthy volunteers at 0.55 T. Figure S2. Simulation results for adiabatic spin‐lock (SL) pulse optimisation. (A) The average preparation efficiency of different candidate adiabatic‐SL pulses with different ωBW and μ. The optimal (ωBW, μ) = (800 Hz, 4) is denoted with the dashed box. (B) Preparation efficiency profile of the optimized adiabatic‐SL pulse. The B0 and B1 design region (B0 off‐resonance range = [−100 100] Hz and B1 factor range = [0.7,1]) is illustrated with the dashed box. Figure S3. bSSFP T2 mapping with 2‐parameter fitting in comparison with T1ρCW and T1ρAd mapping with 3‐parameter fitting. Septal T2 and T1ρ values are shown below the images. T2 (T1ρcw at 0 Hz) map using the proposed sequence with 3‐parameter fitting (41.6 ± 6.8 ms) has lower myocardial T2 values than the bSSFP T2 map with 2‐parameter fitting (51.0 ± 4.8 ms). But T1ρcw at 150 Hz (47.3 ± 8.0 ms) is higher than T1ρcw at 0 Hz using the same 3‐parameter sequence scheme, showing the effect of T1ρ dispersion. Figure S4. B0 and B1 field maps along with AHA plots of mean values and precision for T1ρCW and T1ρAd for the same two volunteers in Figure 3A. Segment‐wise CV (sCV) was also calculated to demonstrate the spatial variability of the T1ρCW and T1ρAd for each subject. Figure S5. Repeatability of myocardial T1ρCW and T1ρAd mapping at each AHA segment in the middle short‐axis slice averaged across all five healthy volunteers. RBS, relative bias. Figure S6. Simulated T1ρ relative errors using different sampling schemes [file MRM-94-1644-s001.docx]

**Supporting Information**

**Sequence simulation experiments**

***Methods***

Additional simulation experiments were performed to evaluate the performance of the proposed sequence for T1ρ mapping with respect to two parameters. First, the maximum SL preparation time is 4$\tau_{HS}$ (60 ms) due to RF hardware constraints. The associated limited dynamic range may influence T1ρ estimation. Thus, different preparation schemes were simulated including (0, 30, 60, SAT), (0, 30 90, SAT), (0, 30, 120, SAT), and a reference scheme using (0, 30, 60, 90, 120, SAT). In simulation, the signal intensity of each SL prepared image was calculated using the following Bloch equation: $S_{i}=e^{-\frac{{\tau_{SL}}_{i}}{T1\rho}}$, while the signal of SAT was considered to be 0 (ideal saturation). Monte Carlo simulations were performed by adding 10,000 times Gaussian noise ($N_{i}$) with a standard deviation of $\sigma$ to $S_{i}$, where the noise level was $\sigma=\frac{1}{SNR}, SNR=40$ according to the approximate SNR level of the in-vivo experiments performed at low-field. The contaminated signals ($S_{i}+N_{i}$) were then used for T1ρ estimation using the proposed 3-parameter fitting methods. Relative errors between estimated and simulated T1ρ values were calculated.

Second, the proposed sequence used a 3 s recovery gap before the second and third SL prepared images. Thus, T1 would influence the recovery of the longitudinal magnetization, and influence the accuracy of T1ρ estimation. In simulation, the entire process of the proposed sequence was simulated using Bloch equation considering incomplete signal recovery and the evolution of signal during bSSFP readout. Saturation efficiency was assumed to be 100%. The simulated signal intensity of the center k-space line was used for T1ρ fitting for each image. Simulations were performed with T1 ranging from 300 to 1100 ms. For each T1 value, Monte Carlo simulations were also performed 10,000 times and T1ρ relative errors were calculated.

***Results***

**Figure S5** shows the T1ρ preparation scheme simulation results. As shown in **Figure S5A,** the adopted sampling scheme (0, 30, 60, SAT) exhibits higher estimation errors than the other schemes for longer T1ρ values. However, it demonstrates good performance for T1ρ < 100 ms with a relative error lower than 1%, which is comparable with the other schemes. Although the adopted sampling scheme has higher relative error than the other schemes at T1ρ > 100 ms, the relative error is within the range of 5 % for T1ρ = 300 ms. As shown in **Figure S5B,** The estimated T1ρ values decrease with longer T1 for all simulated T1ρ. However, for a T1 range of 300 ms to 1100 ms, the T1ρ relative error remains between -5 % to 2% for T1ρ of 100 ms, and does not exceed +/-10 % for a longer T1ρ of 300 ms. For normal myocardium (T1/T1ρ =700/100 ms), the proposed sequence demonstrates excellent performance with T1ρ relative error <0.1%.

**Table S1.** Imaging parameters of conventional 2D T1 and T2 mapping and the proposed T1ρ mapping sequences. MOLLI, modified Look-Locker inversion recovery; bSSFP, balanced steady-state free precession.

|  | **MOLLI T1** | **bSSFP T2** | **T1ρ_CW_** | **T1ρ_Ad_** |
| --- | --- | --- | --- | --- |
| **FOV** | 306×360 mm^2^ | 288×360 mm^2^ | 306×360 mm^2^ | |
| **Resolution (mm^2^)** | 2.3×2.3 | | | |
| **Slice thickness (mm)** | 10 | | | |
| **Readout** | bSSFP | | | |
| **Acceleration** | 2×GRAPPA | | | |
| **Flip angle (°)** | 50 | 70 | 90 | |
| **TR/TE (ms)** | 4.45/1.89 | 4.33/1.8 | 4.45/1.89 | |
| **Bandwidth (Hz/pixel)** | 539 | 558 | 539 | |
| **Lines per readout** | 45 | 48 | 45 | |
| **Preparation pulses** | IR  5(3)3 | T2prep (0/25/55ms) | CW T1ρ(2/30/60ms), SAT | Adiabatic T1ρ(0,2HS,4HS), SAT |
| **Number of images** | 8 | 3 | 4 | 4 |
| **Fitting model** | 3-parameter  inline | 2-parameter  inline | 3-parameter  offline | |

**Table S2.** Phantom T1, T2, T1ρ_CW_ and T1ρ_Ad_ in each vial. MOLLI, modified Look-Locker inversion recovery; bSSFP, balanced steady-state free precession; CV, coefficient of variation; SD, standard deviation.

| **Vial #** | **MOLLI T1** | | **bSSFP T2** | | **T1ρ_CW_** | | **T1ρ_Ad_** | |
| --- | --- | --- | --- | --- | --- | --- | --- | --- |
|  | **Mean [ms]** | **CV [%]** | **Mean [ms]** | **CV [%]** | **Mean [ms]** | **CV [%]** | **Mean [ms]** | **CV [%]** |
| **1** | 430 | 3.0 | 62.9 | 2.3 | 43.9 | 3.2 | 130.3 | 2.9 |
| **2** | 553 | 2.3 | 57.9 | 2.1 | 44.3 | 4.1 | 134.6 | 4.4 |
| **3** | 308 | 1.6 | 66.7 | 3.2 | 42.4 | 5.4 | 113.2 | 3.8 |
| **4** | 887 | 1.6 | 57.2 | 2.9 | 47.6 | 2.7 | 153.0 | 3.1 |
| **5** | 1050 | 1.9 | 57.8 | 2.6 | 48.2 | 3.7 | 148.2 | 4.4 |
| **6** | 723 | 0.9 | 57.3 | 3.1 | 48.4 | 4.3 | 147.2 | 4.1 |
| **7** | 508 | 2.6 | 252.3 | 3.6 | 202.9 | 2.8 | 352.6 | 3.6 |
| **8** | 1427 | 1.3 | 243.8 | 6.7 | 229.7 | 4.4 | 490.2 | 5.6 |
| **9** | 283 | 1.9 | 235.4 | 7.2 | 167.2 | 4.1 | 233.0 | 4.3 |
| **Mean±SD** | - | 1.9±0.7 | - | 3.7±1.9 | - | 3.9±0.9 | - | 4.0±0.8 |

**
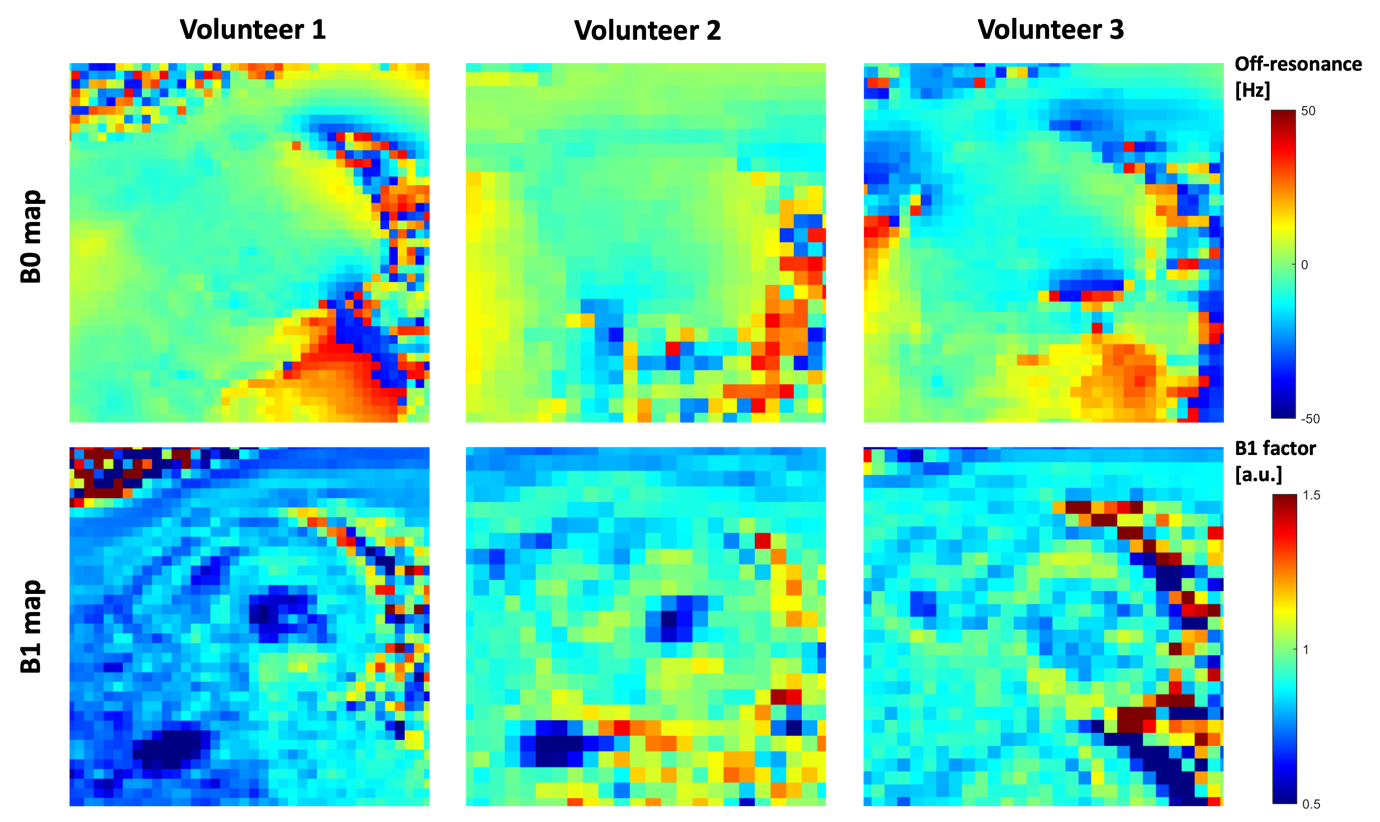
**

**Figure S1.** B0 and B1 maps of three healthy volunteers at 0.55T.

**
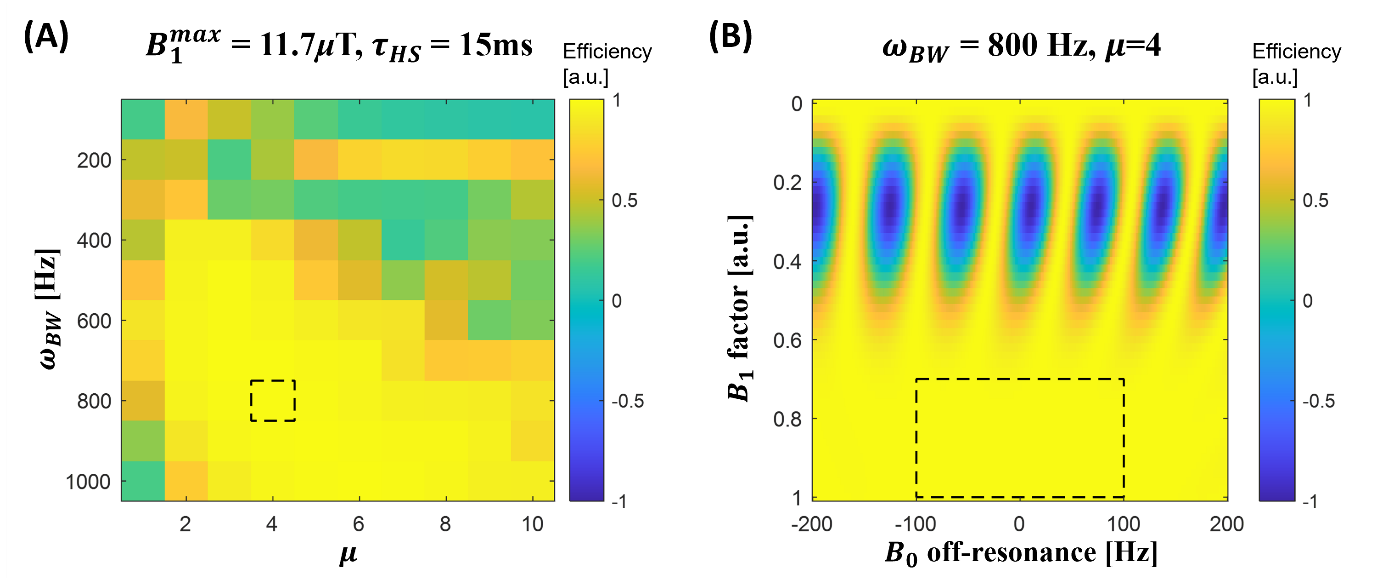
**

**Figure S2.** Simulation results for adiabatic spin-lock (SL) pulse optimisation. (A) The average preparation efficiency of different candidate adiabatic-SL pulses with different $\omega_{BW}$ and $\mu$. The optimal ($\omega_{BW}$, $\mu$)=(800 Hz, 4) is denoted with the dashed box. (B) Preparation efficiency profile of the optimised adiabatic-SL pulse. The B0 and B1 design region (B0 off-resonance range = [-100,100] Hz and B1 factor range = [0.7,1]) is illustrated with the dashed box.

**
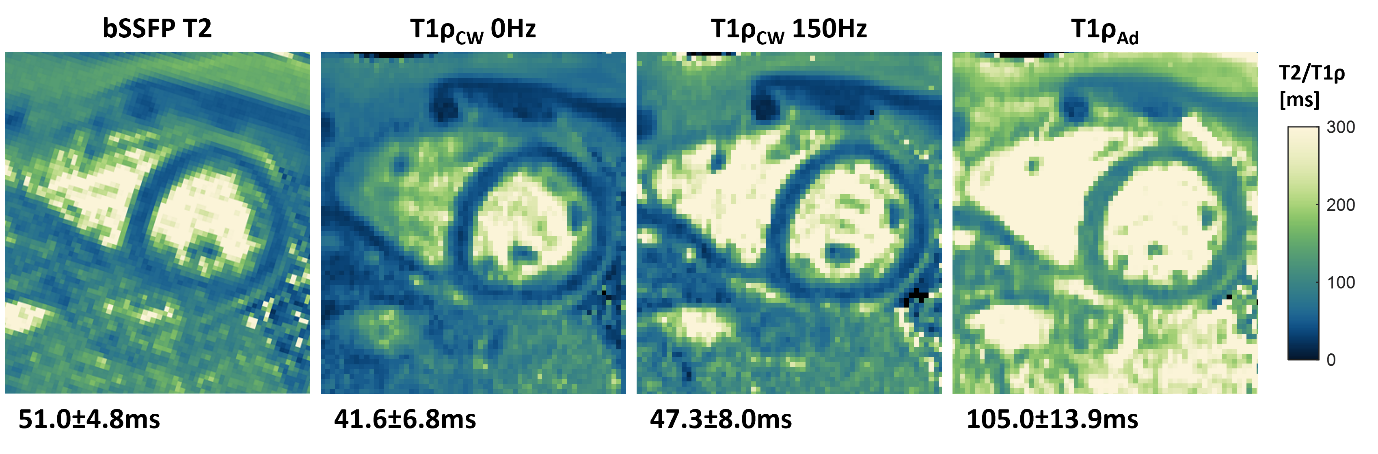
**

**Figure S3.** bSSFP T2 mapping with 2-parameter fitting in comparison with T1ρ_CW_ and T1ρ_Ad_ mapping with 3-parameter fitting. Septal T2 and T1ρ values are shown below the images. T2 (T1ρ_cw_ at 0 Hz) map using the proposed sequence with 3-parameter fitting (41.6 ± 6.8 ms) has lower myocardial T2 values than the bSSFP T2 map with 2-parameter fitting (51.0 ± 4.8 ms). But T1ρ_cw_ at 150 Hz (47.3 ± 8.0 ms) is higher than T1ρ_cw_ at 0 Hz using the same 3-parameter sequence scheme, showing the effect of T1ρ dispersion.


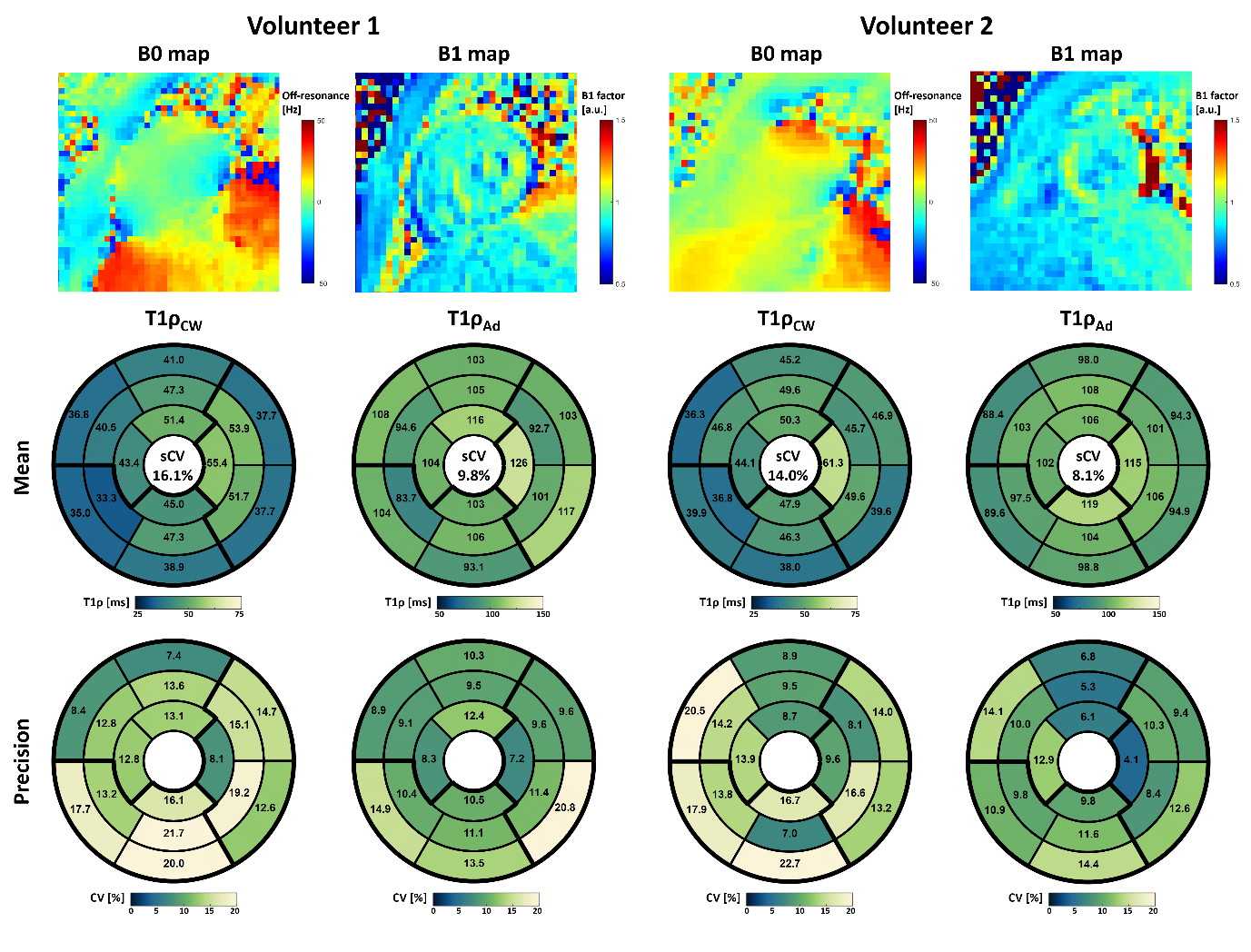


**Figure S4.** B0 and B1 field maps along with AHA plots of mean values and precision for T1ρ_CW_ and T1ρ_Ad_ for the same two volunteers in **Figure 3A**. Segment-wise CV (sCV) was also calculated to demonstrate the spatial variability of the T1ρ_CW_ and T1ρ_Ad_ for each subject.

**
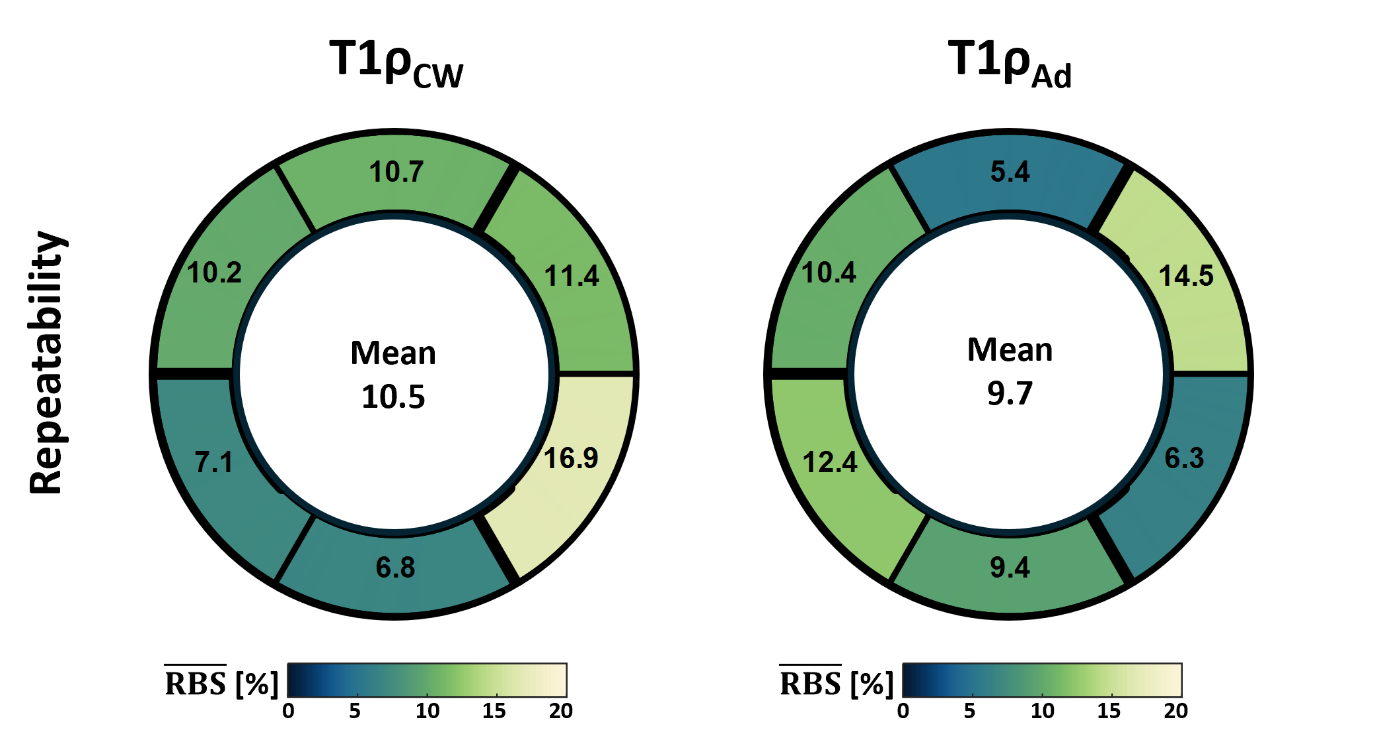
**

**Figure S5.** Repeatability of myocardial T1ρ_CW_ and T1ρ_Ad_ mapping at each AHA segment in the middle short-axis slice averaged across all five healthy volunteers. RBS, relative bias.

**
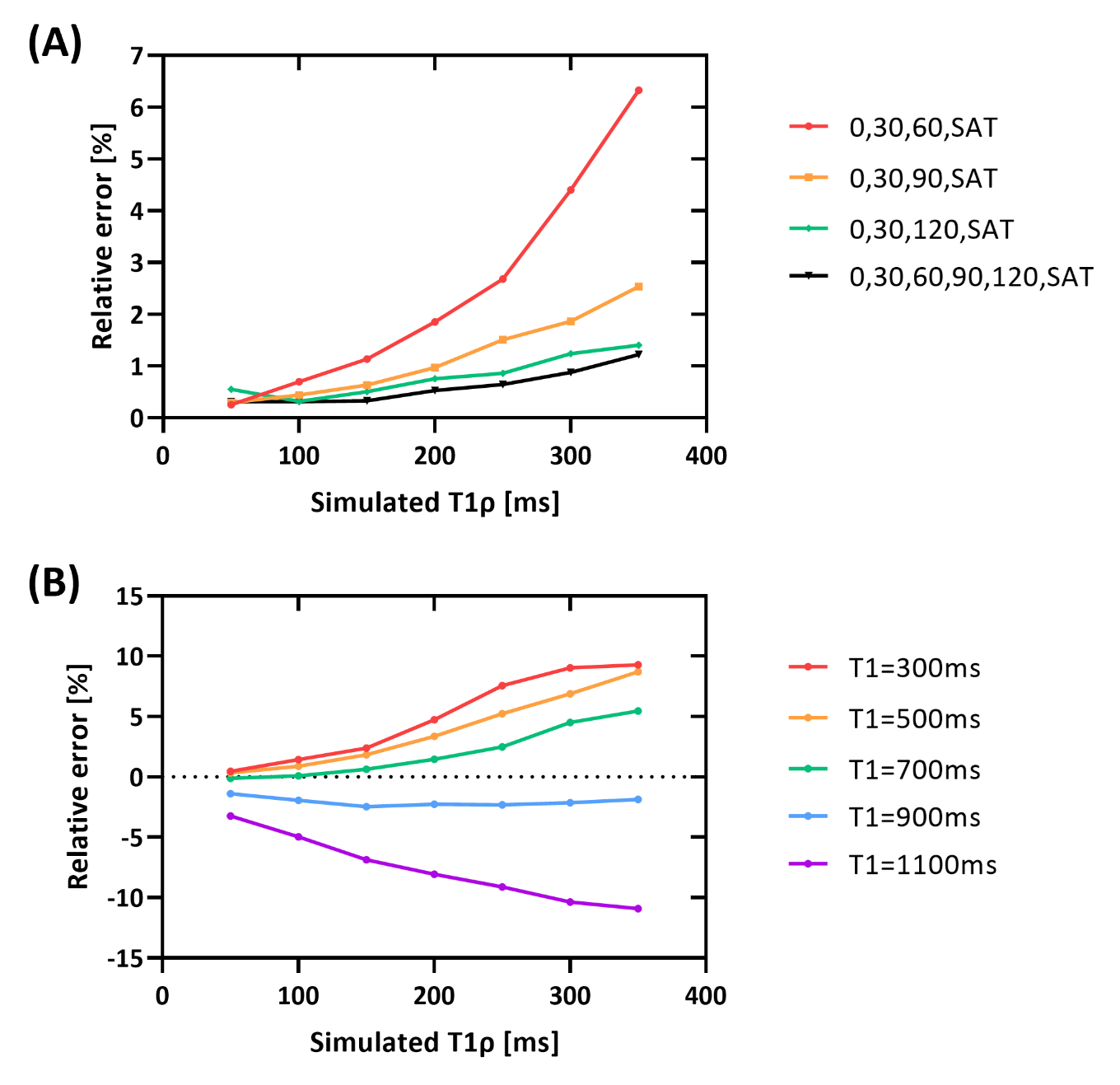
**

**Figure S6.** Simulated T1ρ relative errors using different sampling schemes (A) and using the proposed sampling scheme for different T1 values (B).
